# CircRNA_100395 Carried by Exosomes From Adipose-Derived Mesenchymal Stem Cells Inhibits the Malignant Transformation of Non-Small Cell Lung Carcinoma Through the miR-141-3p-LATS2 Axis

**DOI:** 10.3389/fcell.2021.663147

**Published:** 2021-03-25

**Authors:** Chong Zhang, Jinlin Cao, Wang Lv, Haibo Mou

**Affiliations:** ^1^Department of Thoracic Surgery, The First Affiliated Hospital, School of Medicine, Zhejiang University, Hangzhou, China; ^2^Department of Medical Oncology, Shulan (Hangzhou) Hospital, Affiliated to Shulan International Medical College, Zhejiang Shuren University, Hangzhou, China

**Keywords:** non-small cell lung carcinoma (NSCLC), exosomes from adipose-derived mesenchymal stem cells, Hippo/YAP signalling pathway, circRNA_100395, miR-141-3p

## Abstract

Objective: The specific purpose of this study is to investigate the impact exosomes from adipose-derived mesenchymal stem cell (AMSC) has on non-small cell lung carcinoma (NSCLC) and the relative applications. Methods: circ_100395, miR-141-3p, and LATS2 were expressed and detected in NSCLC and paracancerous tissues as well as NSCLC cell lines. Pearson correlation analysis, Dual-Luciferase Reporter Assay and RNA pull-down assay were used to validate their expression and interaction, respectively. After isolation and culture of AMSCs, exosomes were extracted and identified. EdU, epithelial-mesenchymal transition (EMT), and cell colony formation assay were used to distinguish the biological activity of the cells. Expression Hippo/YAP signalling pathway-related proteins were measured by western blotting. Subsequently, tumour volume and weight were confirmed based on xenograft nude mice models, Ki-67 and LATS2 expression was observed by immunohistochemistry. Results: circ_100395 was lowly expressed in NSCLC tissues or cells. The negative correlations and interactions were confirmed between circ_100395 and miR-141-3p, miR-141-3p, and LATS2. AMSC-derived exosomes with overexpression of circ_100395 (exo-circ_100395) significantly inhibited the biological activity as well as EMT of H1650 cells and Hippo/YAP signalling pathway activity. In addition, exo-circ_100395 markedly reduced tumour volume and weight as well as Ki-67 and LASP1 expression *in vivo*. However, overexpressed miR-141-3p or knocked down LATS2 alleviated the above effects. Conclusion: Exo-circ_100395 can increase LATS2 expression by sponging miR-141-3p to regulate Hippo/YAP signalling pathway, thereby inhibiting NSCLC malignant transformation.

## Introduction

One of the leading risk factors causing death in cancer patients particularly within industrial countries is non-small cell lung carcinoma (NSCLC). NSCLC accounts for about 85% of all existing lung cancers with a below 15% survival rate (Bunn et al., 2003; Siegel et al., 2017). Regular subtypes relating to NSCLC are adenocarcinoma, large cell carcinoma and squamous cell carcinoma (Bray et al., 2018). Clinically, the current prime method of therapy for NSCLC involves surgical resection, chemotherapy and radiotherapy. However, therapeutic effects on NSCLC is very limited. NSCLC patients cannot be cured by these methods and the prognosis is also poor (Jemal et al., 2011). In addition, inhibiting angiogenesis as a treatment for NSCLC has existed and utilised in clinical testing for several years (Abéngozar et al., 2012). However, the transmission of cytotoxic drugs to lesions can be weakened by angiogenesis inhibitors, therefore resulting in affected therapeutic effects of the relating anti-tumour drugs (Van der Veldt et al., 2012). Conley et al. have also pointed out that antiangiogenic agents can stimulate tumour cells invasive ability which allows it to additionally adjust to harmful microenvironments (Conley et al., 2012). Therefore, revaluation and consideration will be needed in regards to antiangiogenic treatment. Thereby, finding new treatments for NSCLC is urgently needed.

Extensive studies have been conducted on mesenchymal stem cells (MSCs have a variety of characteristics, including low immunogenicity, multilineage differentiation, and promotion of tissue regeneration. The safety of these have gone under assessment via conducting clinical experimentation in 1995 (Lazarus et al., 1995), and ever since, treatments based on MSCs for cancers have gained wide consideration (Ramdasi et al., 2015). Among them, numerous studies have paid more attention to adipose-derived mesenchymal stem cells (AMSCs) in malignant cells. For instance, Pendleton et al. (2013) have revealed the potential AMSC has to treat glioma. Lou et al. (2015) have discovered AMSC-derived exosomes (AMSC-exo) can increase the sensitivity of cancer cells in the liver has for chemotherapy. Exosomes, small vesicles with a bilayer membrane between 30 and 150 nm in diameter, have been demonstrated to be implicated in a variety of diseases progressions (Valadi et al., 2007). Exosomes are a special class of carriers for signal transmission, which can transport miRNAs, circRNAs, and other signalling molecules into target cells or organs, thus playing a regulatory role (Thomou et al., 2017). Liu et al. (2020) confirmed that exosome-derived miR-522-3p shed by EGFR-TKI–resistant NSCLC cells carrying T790M mutation can induce gefitinib resistance in sensitive cell via activating PI3K/AKT signalling pathway. Kim et al. (2020) showed that NSCLC cell-derived exosomal miR-619-5p promotes tumor angiogenesis and metastasis. Nevertheless, there are relatively few studies regarding the impact AMSC-exo has on NSCLC progression.

Circular RNAs (circRNAs), in recent years, have been proved to be critical information transfer molecules and main biomarkers during the growth of cells and development of tumour (Ashwal-Fluss et al., 2014). Research has indicated that circRNA_100395 (circ_100395) is downregulated in lung cancer tissues and can inhibit lung cancer progression by regulating miR-1228/TCF21 pathway (Chen et al., 2018). Moreover, Chen et al. (2019) have reported that up-regulation of circ_100395 in liver cancer can inhibit cell proliferation, induce apoptosis, and silence epithelial-mesenchymal transition (EMT) pathway. And up-regulated circ_100395 present in ovarian cancer can regulate the miR-1228/p53/EMT axis, thereby inhibiting tumour growth and metastasis (Li et al., 2020). However, the relationship between AMSC-exo delivered circ_100395 and NSCLC has not been reported.

Here, we extracted AMSC-exo to explore the effect of circ_100395 on *in vitro* and vivo NSCLC development and its mechanism, hence bringing new ways for NSCLC therapy.

## Materials and Methods

### Tissue Specimen Collection

A grand sum including 60 NSCLC tissues (NSCLC group) duos as well as their adjoined regular tissues (normal group) was gained using surgical collection method. These specimens were taken from NSCLC patients treated in our hospital from June 2016 to December 2018. With informed consent from each patient with approval for research from the Clinical Experimental Ethics Committee of our hospital and strictly following the Declaration of Helsinki.

### Cell Culture and Treatment

#### Isolation, Culture and Treatment of Mesenchymal Stem Cells That Are Adipose-Derived

Human subcutaneous adipose tissue was acquired from patients undergoing abdominal liposuction in The First Affiliated Hospital, Zhejiang University School of Medicine. Adipose tissue was processed as previously described [10]. After a three time rinse of sterile PBS under sterile conditions, ophthalmic scissors was used to slice up the tissue into 1 mm^3^ blocks. Subsequently, 0.075% type I collagenase was added, followed by digestion at 37°C and 150 rpm for 1 h. DMEM/F12 medium including 10% fetal bovine serum was utilised to terminate digestion. Next, centrifugation (1,000 g, 10 min) was performed, and then the supernatant was aspirated. For resuspension of cells, fresh DMEM/F12 medium was added. Finally, cells were transferred to a 25 cm^2^ culture flask and cultured at 37°C with 5% CO_2_ (Thermo, United States). AMSCs after 3rd passages were used for subsequent experiments.

According to instructions of lipo2000 (Thermo, United States), AMSCs were transfected with circ_100395 overexpression vector (circ_100395 vector) and its empty vector (NC vector), which were cloned and integrated by Shanghai Sangon Biotech (Shanghai, China). AMSC-NC group and AMSC-circ_100395 group was the name used for transfected AMSCs, respectively.

#### Culture of NSCLC and Human Bronchial Epithelial Cells Cell Lines

Shanghai Institute of Biological Sciences, Chinese Academy of Sciences (Shanghai, China) provided A549, H1650, H460, H1299 from the NSCLC cell line along with 16 HBE-T from a cell line of human bronchial epithelial cell. With conditions of 37°C and 5% CO_2_, the cell culture was performed within RPMI 1640 medium which contained supplementary 10% fetal bovine serum. Subsequent experiments were conducted on cells with good growth status that were within the logarithmic growth phase. Guangzhou RiboBio Co., Ltd. (Guangzhou, China) provided H1650 cells were transfected with circ_100395 vector and NC vector, circ_100395 siRNA and miR-141-3p mimics and inhibitor along with their negative control. The transfected H1650 cells identified as circ_100395 group, NC group, si-circ_100395 group, si-NC group, miR-141-3p group, mi-NC group, in-miR-141-3p group, and in-NC group.

### Extraction and Identification of Exosomes

AMSC-exo were extracted by GS^TM^ Exosome Isolation Reagent (Geneseed, China) from each treatment group, named exo-NC, exo-circ_100395. H1650 cells were treated with PBS, two exosomes, exo-circ_100395 + miR-141-3p mimics, and exo-circ_100395 + si-LATS2, respectively, which were named as PBS, exo-NC, exo-circ_100395, exo-circ_100395 + miR-141-3p, and exo-circ_100395 + si-LATS2 groups.

After negative staining of exosomes by uranyl acetate, a transmission electron microscope was used to observe exosome morphology. Nanoparticle tracking analysis was utilised to verify exosome diameter while The western blotting was done to assess CD9, CD63, and TSG101 exosome marker proteins that are expressed.

### qRT-PCR

By using TRizol method, extraction of total RNA was gained, this following the use of NanoDrop in order to determine RNA mass and clarity. Random primer reverse transcription kit (Thermo, United States) was used to reverse transcribe RNA to cDNA. SYBR GREEN kit (TaKaRa, Japan) was adopted for qRT-PCR. The qRT-PCR condition guidelines are 95°C, 2 min, with 40 cycles of 95°C, 15 s, 60°C, 1 min and 72°C, 30 s. A total of six replicates were set up for this experiment. 2^–ΔΔCt^ method with U6 and β-actin was used for referencing internally in quantitative analysis. Table 1 illustrates the primer sequences that were used.

**TABLE 1:** Primer sequences.

**Gene**	**Sequences (5′ to 3′)**
circ_100395	F: AGTGATGTGGCCCCTACA AG
	R: CCACTGGAGACCACTGGTTG
miR-141-3p	RT: GTCGTATCCAGTGCGTGTCGTGGAGT CGGCAATTGCACTGGATACGACCCATCT
	F: CCTCGTCTTGAGCTGAGAGC
	R: AGGGCTCCCTGAAGGTTACT
β-actin	F: GCATGGGTCAGAAGGATTCCT
	R: TCGTCCCAGTTGGTGACGAT
U6	F: CTCGCTTCGGCAGCACA
	R: AACGCTTCACGAATTTGCGT

### 5-Ethynyl-2′-Deoxyuridine (EdU) Analysis

Transfected cells were plated in 24-well plates and stained according to the instructions of EdU staining kit (Thermo, United States). After mounting step, fluorescence microscope (model: FM-600, Shanghai Puda Optical Instruments Co., Ltd.) was used to view the cells for 6–10 fields, therefore, recording the number of positive cells that are present in individual fields. EdU labelling rate (%) = NO. of positive cells/(NO. of positive + negative cells) × 100%.

### Cell Colony Formation Assay

On completion of digestion using 0.25% trypsin, resuspension was completed using RPMI 1640 complete medium containing 0.35% agarose for transfected cells. Subsequently, 6-well plates containing 0.6% agarose was plated with 1 × 10^5^ cells individually, followed by cell culture at 37°C with 5% CO_2_. Termination of the culture was applied when colonies could be observed without instrumental help. An inverted microscope was used to photograph and count the number of colonies present following staining using 0.1% crystal violet solution.

### Flow Cytometry

On completion of digestion using trypsin, the cells were placed into centrifuge tubes with trypsin, followed by a two time rinse using precooled sterile PBS. Cell massed was then modified to 5 × 10^5^ cells/mL. A total of 10 μL of Annexin V-FITC and 20 μg/mL PI solution respectively were incorporated into the 200 μL cell suspension. The suspension was then incubated for 10 min at room temperature and avoiding light. Lastly, incorporation of 500 μL PBS was done before flow cytometer was adopted for apoptosis detection.

### Transwell Invasion Assay

The Matrigel gel-coated transwell upper chamber was left to stand for about 30 min at 37°C with 5% CO_2_. Subsequently, incubation for 12–24 h t 37°C in 5% CO_2_ was conducted via incorporated 300 μL cell suspension and 700 μL of 20% fetal bovine serum within upper and lower chambers, respectively. After that, PBS was used to rinsed through the insert three times before fixation with 1% glutaraldehyde and staining by 0.1% crystal violet for 12 h. On completion of staining, cells were again washed with PBS before drying. Finally, an observation under an upright microscope for random 6–10 fields was carried out for documentation of the quantity of positive cells present in individual sections.

### Wound Healing Assay

After transfection of the cells plated in 6-well plates, a 10 μL sterile tip was used to apply scratches perpendicular to the horizontal plane in individual wells. PBS was employed to remove cell debris and detached cells. After that, with the addition of fresh medium, and incubation for 24 h were performed. Finally, an inverted microscope was used to capture and document the outcomes of the experiment. The scratched area was measured by Image J software.

### Western Blot

Following extraction of total protein using cell lysate, a BCA kit was used to establish the concentration of extracted protein. Subsequently, 1× loading buffer was used to gain denaturation of 20 μg of protein accomplished by boiling. SDS-PAGE was then used to isolate proteins before being transported to PVDF membranes and blocked by the use of 5% fat-free dry milk for 1-h. Following this was an all-night incubation of the membranes 4°C with primary antibodies E-cadherin, vimentin, N-cadherin, LATS2, p-YAP, and YAP (Abcam, United Kingdom). After washing the membranes for three times, another 1-h incubation was carried out with secondary antibodies at room temperature. On completion of incubation, the membranes were washed for three times. Finally, the proteins were developed by chemiluminescence reagents. The images were collected using a gel imaging system with ImageJ software used to analyse protein band grey values. GAPDH was then used as a reference internally for determination of relative expressed protein.

### Dual-Luciferase Reporter Assay

StarBase v3.0 and TargetScan were used to foretell of circ_100395 with miR-141-3p and miR-141-3p with LATS2 binding sites correspondingly. Insertion of wild-type (WT) mutant (MUT) sequence binding sites of the respectively were done to the downstream of the firefly luciferase gene to construct expression vectors (Promega, USA). By using Lipo2000, recombinant plasmids consisting of miR-141-3p mimics and pmirGLO-circ_100395-WT/MUT was used to transfect 293T cells, recombinant plasmids consisting of miR-141-3p mimics and pmirGLO-LATS2-WT/MUT recombinant, and control plasmids, respectively. After 48 h of transfection, the dual-luciferase reporter assay kit was utilised in order for quantification of luciferase activity.

### RNA Pull-Down Assay

A total of 50 nM biotin-conjugated miR-141-3p or miRNA NC was used for transfection of H1650 cells for 48 h, followed by rinsing with PBS for three times. Subsequently, RNase-free BSA and yeast tRNA was used to pre block M-280 streptavidin magnetic beads while cells underwent lysis with the incorporation of a cell lysis buffer. The lysis buffer from both groups was collected and the supernatant obtained by centrifuging for 10 min at 14,000 rpm under 4°C conditions. The supernatant was separated into two with one as Input group and the other incorporated alongside beads and went under incubation for 3 h under 4°C conditions. The magnetic beads coated with biotin-conjugated RNA were then washed and RNA was resuspended and extracted by using TRIzol reagent. Finally, qRT-PCR was used to measure the content of the target genes was by and the test was run another 3 trials.

### Establishment of Xenograft Models

Fifteen nude mice weighing 18–23 g was separated into five groups of three in random order. Injection of 100 μL H1650 suspension subcutaneously was applied to the right axilla of nude mice. Injected mice were named as PBS group, exo-NC group, exo-circ_100395 group, exo-circc_100395 + miR-141-3p group, and exo-circ_100395 + si-LATS2 group. Tumour size (volume = 0.5 × length × width^2^) was measured weekly from the time of subcutaneous injection. Four weeks later, after 10% chloral hydrate was injected intraperitoneally for anesthesia, the mice were put to death using cervical dislocation with tumours removed. Based on the approval of the ethics committee, this trial was in accordance with animal ethics.

### Immunohistochemistry

After being routinely treated with paraffin embedding, sectioning, deparaffinisation, heat-induced epitope retrieval, inactivation of peroxidase, PBS was used to immersed tumour tissues from mice with an incubation alongside primary antibodies Ki-67 and CD34 at 37°C surroundings for 1 h, followed by another round of PBS immersion. Next, incubation accompanied by secondary antibodies at 37°C were performed, following additional immersion in PBS. Following development by DAB, the samples were counterstained with hematoxylin. Then after being dehydrated in ethanol and clearing, the tissues were mounted with neutral balsam. Finally, the positive expression of proteins in tumour tissues was observed under a microscope.

### Statistics

SPSS 26.0 was used to conduct a single-way evaluation of variance and individual specimens *T*-test. Mean ± standard deviation (SD) was used to represent outcomes. Conduction of the Pearson correlation analysis was done with significant differences being suggested with *p* < 0.05 results.

## Results

### Low Expression of circ_100395 in NSCLC Tissues and Cells

**FIGURE 1:**
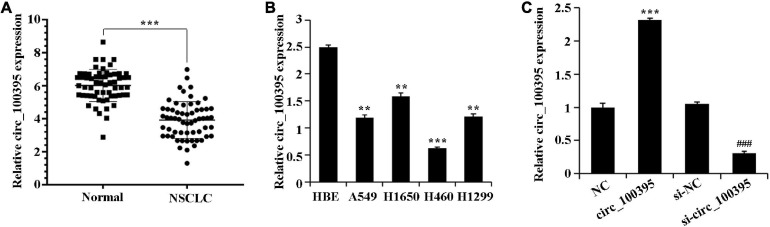
Low circ_100395 expressed in NSCLC tissues and cells. **(A)** Evaluation of circ_100395 expressed in paracancerous and NSCLC tissues using qRT-PCR. ***p < 0.001 vs Normal group. **(B)** Evaluation of circ_100395 expressed in human bronchial epithelial cell line 16 HBE-T and NSCLC cell lines using qRT-PCR. **p < 0.01 and ***p < 0.001 vs HBE; **(C)** Evaluation of circ_100395 expressed in H1650 cells of each treatment group using qRT-PCR. ***p < 0.001 vs NC group and ^###^*p* < 0.001 vs si-NC group.

By detecting expressed circ_100395 within 60 NSCLC and paracancerous tissue pairs, it can be seen that the expressed circ_100395 levels were dropped remarkably NSCLC tissues (Figure 1A). Additional analysis at the cellular level also revealed a significant reduction of circ_100395 expression in NSCLC cell lines (Figure 1B), with the highest expression in H1650 cells. Therefore, H1650 cells were used for subsequent experiments.

To determine the functions of circ_100395, transfection of H1650 cells were done using circ_100395 vector and circ_100395 siRNA, and the transfection efficiency was confirmed by qRT-PCR. Circ_100395 was significantly increased after overexpression of circ_100395, while it was markedly decreased after the interference of circ_100395 (Figure 1C).

### Circ_100395 Inhibits NSCLC Development by the Inhibition of Hippo/YAP Signalling Pathway

**FIGURE 2:**
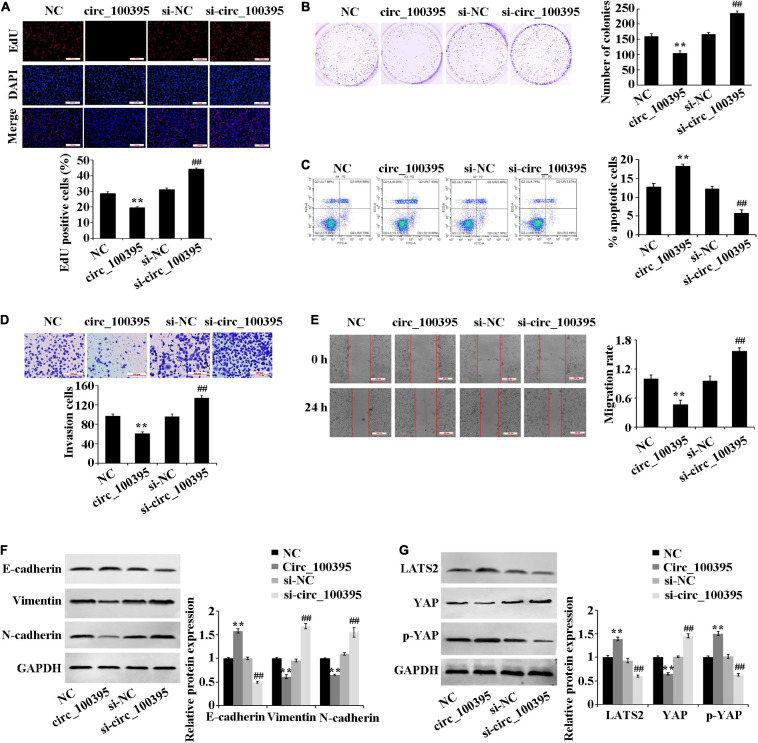
Outcomes circ_100395 has regarding the progression of NSCLC cells and Hippo/YAP signalling pathway. **(A)** Cell proliferation identified by using an EdU assay-base; **(B)** Cell viability identified by using a cell colony formation assay-base; **(C)** Flow cytometry-based detection of cell apoptosis; **(D)** Cell invasion identified by using a Transwell assay-base; **(E)** Wound healing assay-assessment of cell migration; **(F)** E-cadherin, vimentin, and N-cadherin proteins identified by using Western blot of; **(G)** LATS2, p-YAP, and YAP proteins identified by Western blot. **p < 0.01 vs NC group; ^##^*p* < 0.01 vs si-NC group.

Overexpressed circ_100395 has remarkably decreased proliferation, cell viability, invasion and migration of H1650 cells. It can also cause increased apoptosis, at the same time interference of circ_100395 achieved the opposite effects (Figures 2A–E). These results confirmed that circ_100395 is able to inhibit metastasis and proliferation in NSCLC cells.

Additional inspection of expressed EMT marker proteins and Hippo/YAP signalling pathway-related proteins, which were associated with tumour metastasis was carried out. Compared with the NC group, the E-cadherin protein expression was significantly increased, and the protein expression of vimentin and N-cadherin were reduced in the circ_100395 group. In addition, upregulation of circ_100395 resulted in a higher expression of LATS2 and YAP phosphorylation, and a lower expression of YAP. However, interference of circ_100395 led to contrasting effects in the expression of the above-related proteins (Figures 2F,G). Therefore, circ_100395 inhibits NSCLC development and blocks the Hippo/YAP signalling pathway.

### Circ_100395 Functioning as miR-141-3p Sponge

**FIGURE 3:**
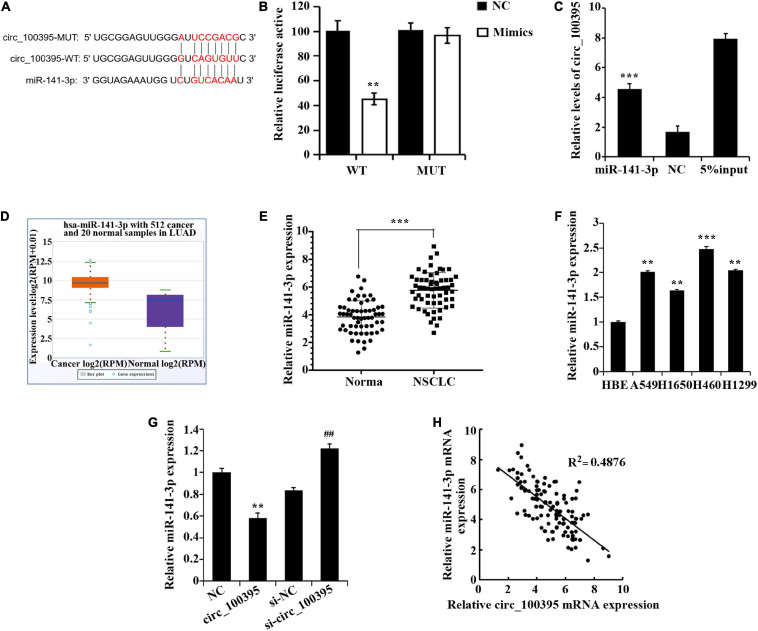
circ_100395 can as a sponge to adsorb miR-141-3p. **(A)** The predicted targeting sequence of circ_100395 and miR-141-3p. **(B)** Dual-luciferase reporter assay-based validation that circ_100395 targets miR-141-3p. **p < 0.01 vs NC group. **(C)** RNA pull down assay-based validation that circ_100395 targets miR-141-3p. ***p < 0.001 vs NC group. **(D)** MiR-141-3p expression in non-small cell lung cancer (NSCLC) tissues in StarBase3.0 database; **(E)** qRT-PCR detection of miR-141-3p expression in NSCLC tissues and paracancerous tissues. ***p < 0.001 vs Normal group. **(F)** qRT-PCR detection of miR-141-3p expression in NSCLC cell lines and human bronchial epithelial cell line 16 HBE-T. **p < 0.01 and ***p < 0.001 vs HBE. **(G)** qRT-PCR detection of miR-141-3p expression in H1650 cells after overexpression and interference of circ_100395. **p < 0.01 vs NC group, ^##^*p* < 0.01 vs si-NC group. **(H)** A negative correlation between circ_100395 and miR-141-3p expression in NSCLC tissues.

Dual-luciferase reporter assay and RNA pull down assay were performed to confirm that circ_100395 binded to miR-141-3p (Figures 3A–C). Excessive miR-141-3p was expressed in NSCLC tissues discovered through the StarBase3.0 database (Figure 3D), which was also confirmed by qRT-PCR (Figure 3E). Expressed miR-141-3p was also notably risen in NSCLC cell lines (Figure 3F).

Additionally, in H1650 cells, overexpression of circ_100395 led to the down-regulation of miR-141-3p expression, while interference of circ_100395 induced an increase of it (Figure 3G). A contrary relationship was found amidst expressed circ_100395 and miR-141-3p within tissues of NSCLC when analysed using Pearson correlation analysis (Figure 3H). Collectively, circ_100395 could competitively bind to and regulate miR-141-3p in cells of NSCLC.

### Circ_100395 Up-Regulates LATS2 Expression by Competitively Binding to miR-141-3p

**FIGURE 4:**
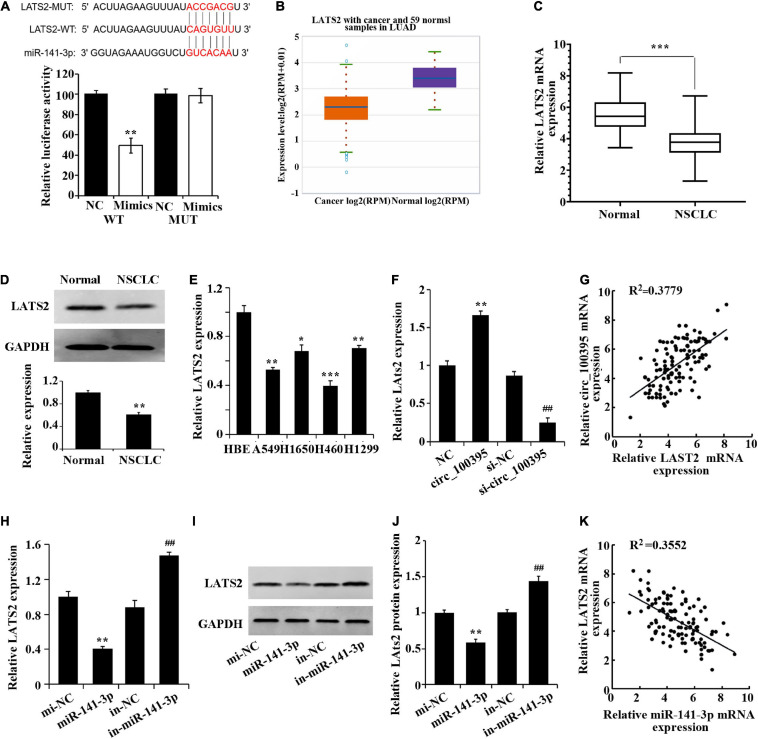
Circ_100395 up-regulates LATS2 expression by competitively binding to miR-141-3p. **(A)** Confirmation of miR-141-3p targeting LATS2 gene via Dual-luciferase reporter assay. **(B)** LATS2 expression in NSCLC tissues in the StarBase3.0 database; **(C)** LATS2 expressed in paracancerous and NSCLC tissues qRT-PCR analysis. **(D)** LATS2 protein expressed in paracancerous and NSCLC tissues detected via western blot; *p < 0.05, **p < 0.01, and ***p < 0.001 vs Normal group. **(E)** Expressed LATS2 in human bronchial epithelial cell line 16 HBE-T and NSCLC cell lines via qRT-PCR analysis, *p < 0.05, **p < 0.01, ***p < 0.001 vs HBE. **(F)** qRT-PCR analysis of LATS2 expression in H1650 cells after overexpression and interference of circ_100395; **p < 0.01 vs NC group, ^##^*p* < 0.01 vs si-NC group. **(G)** A beneficial relationship was found amidst expressed circ_100395 and LATS2 within tissues of NSCLC; **(H)** Expressed LATS2 post overexpression and interference of miR-141-3p discovered using qRT-PCR. **p < 0.01 vs NC group, ^##^*p* < 0.01 vs si-NC group. **(I,J)** Expressed LATS2 protein in H1650 cells post overexpression and interference of miR-141-3p discovered using Western blot. **p < 0.01 vs NC group, ^##^*p* < 0.01 vs si-NC group; **(K)** A contrary relationship was found amidst expressed miR-141-3p and LATS2 within tissues of NSCLC.

A prognosis stating LATS2 as a targeted gene of miR-141-3p by the bioinformatics website was again established dual-luciferase reporter assay (Figure 4A). Low expression of LATS2 was found in NSCLC tissues through StarBase 3.0 database (Figure 4B), which was also proved via the use of qRT-PCR and western blotting (Figures 4C,D). LATS2 expression in NSCLC cell lines also notably dropped (Figure 4E).

Additionally, in H1650 cells, overexpression of circ_100395 led to the up-regulation of LATS2 expression, while interference of circ_100395 induced a decrease of LATS2 expression (Figure 4F). A beneficial relationship was found amidst expressed circ_100395 and LATS2 within tissues of NSCLC when analysed using Pearson correlation analysis (Figure 4G).

A remarkable fall in expressed LATS2 in miR-141-3p group was gained from qRT-PCR and western blot, while markedly increased in in-miR-141-3p group (Figures 4F–J). A contrary relationship was found amidst expressed miR-141-3p and LATS2A tissues of NSCLC (Figure 4K). Combined with the previous result, in NSCLC cells circ_100395 could competitively bind to miR-141-3p, thereby up-regulating LATS2 expression.

### Exosomes From AMSCs Transport circ_100395 Into NSCLC Cells

**FIGURE 5:**
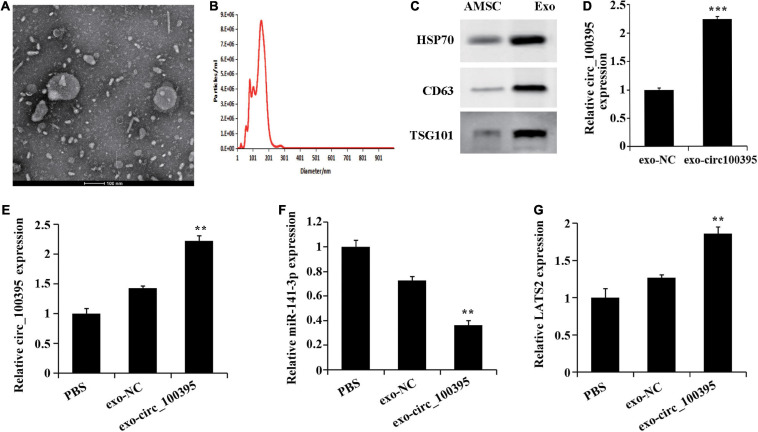
AMSCs exosomes transport circ_100395 into NSCLC cells. **(A)** Morphology of AMSC observed using transmission electron microscopy (100 nm); **(B)** AMSC size analysed using nanoparticle tracking**; **(C)** Expressed HSP70, CD63, and TSG101 detected via western blot; **(D)** Exosomes in circ_100395 analysed via qRT-PCR; **(E–G)** Expressed circ_100395, miR-141-3p, LATS2 in H1650 cells post exo-NC or exo-circ_100395 treatment analysed via qRT-PCR. **p < 0.01 and ***p < 0.001 vs exo-NC group.

Previous research has confirmed absorption and fusion relating to MSC-derived exosomes by cancer cells [17], and their ability to transport RNA molecules (mRNA, miRNA, and circRNA) [15, 18]. Therefore, we speculated that AMSC-exo can be used as an effective carrier to transport circ_100395, thus inhibiting NSCLC development. By collecting AMSC-exo, round or oval vesicles with membranous structure could be observed under the transmission electron microscope (Figure 5A). Their diameter ranged from 87 to 141 nm, with an average diameter of 116 nm (Figure 5B). Meanwhile, HSP70, CD63, and TSG101 expressed protein levels notably risen in the exosomes compared with AMSC (Figure 5C). Collectively, AMSC-exo was successfully acquired.

Further, exosomes were collected from AMSCs transfected with circ_100395 vector, named exo-circ_100395. The expression of circ_100395 in the exo-circ_100395 group was found to be notably elevated end compared with exo-NC group (Figure 5D). In addition, in H1650 cells, an increase of LATS2 expression, and a decrease of miR-141-3p expression were also revealed in exo-circ_100395 group when compared with exo-NC group (Figures 5E–G).

### Circ_100395 Carried by Exosomes From AMSCs Inhibiting NSCLC Progression Through the miR-141-3p/LATS2 Axis

**FIGURE 6:**
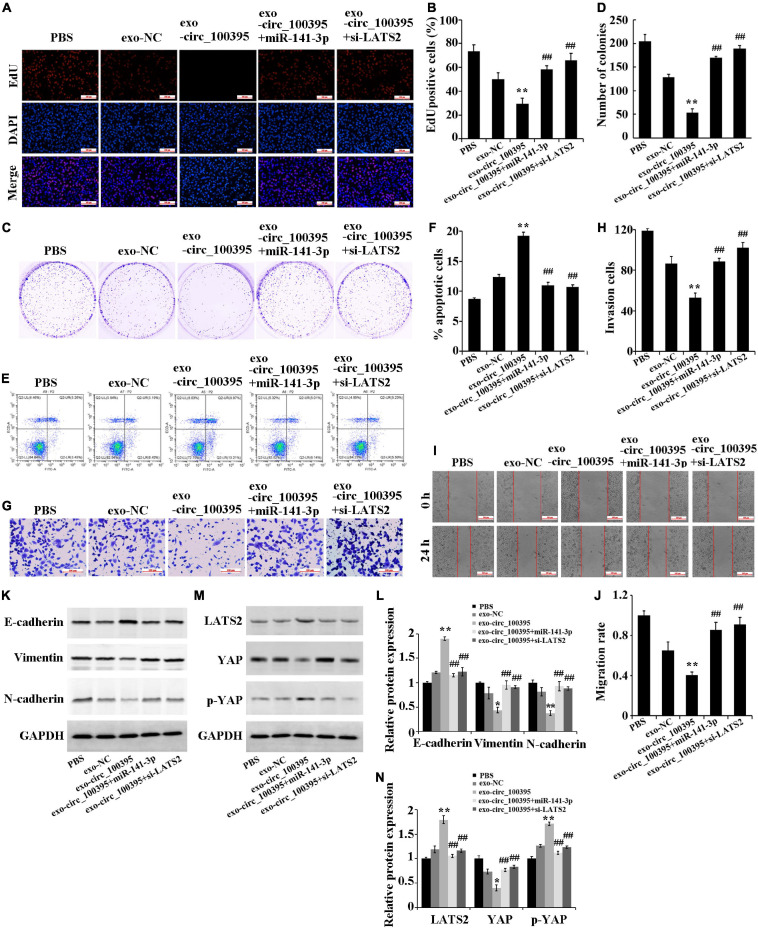
Circ_100395 carried by exosomes from AMSCs inhibiting NSCLC progression through the miR-141-3p/LATS2 axis. **(A,B)** EdU assay-based observation of cell proliferation; **(C,D)** Colony formation assay-based observation of cell viability; **(E,F)** Flow cytometry-based detection of cell apoptosis; **(G,H)** Cell invasion exposed via transwell assay; **(I,J)** Wound healing assay-based detection of cell migration; **(K,L)** Detecting expressed E-cadherin, vimentin, and N-cadherin protein in individual treatment groups via western blot; **(M,N)** Detecting expressed LATS2, p-YAP, and YAP protein in individual treatment groups via western blot. **p < 0.01 vs exo-NC group, ^##^*p* < 0.01 vs exo-circ_100395 group.

Further, we determined the functional effects of circ_100395 delivered by AMSC-exo on NSCLC. The result figured out that AMSC-exo could carry circ_100395 to H1650 cells, thereby significantly reducing cell proliferation, cell viability, invasion and migration, and increasing the apoptotic rate. Compared with the exo-circ_100395 group, the proliferation level, cell viability, invasion and migration of cells in the exo-circ_100395 + miR-141-3p, and exo-circ_100395 + si-LATS2 groups were marked increased, and the apoptotic rate was decreased (Figures 6A–J). In addition, exo-circ_100395 significantly promoted the expression of E-cadherin, LATS2 and p-YAP, and inhibited the protein expression of vimentin, N-cadherin and YAP. However, compared with exo-circ_100395 group, the expression of the above proteins in group exo-circ_100395 + miR-141-3p and exo-circ_100395 + si-LATS2 was reversed (Figures 6K–N). Overall, these results revealed that exo-circ_100395 could inhibit NSCLC procession through regulating the miR-141-3p/LATS2 axis.

### Circ_100395 Carried by Exosomes From AMSCs Inhibiting NSCLC Growth of *in vivo*

**FIGURE 7:**
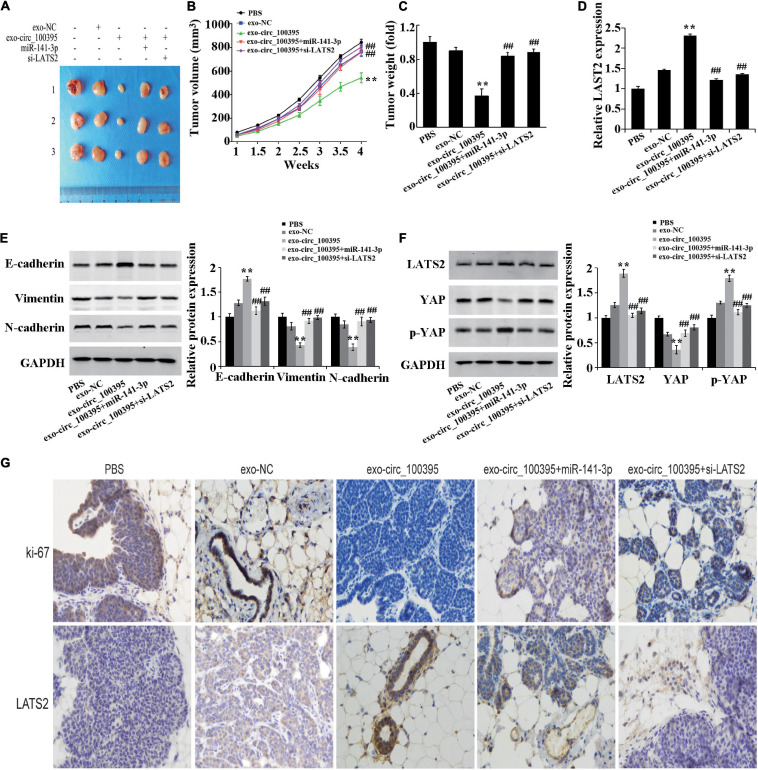
Circ_100395 carried by exosomes from AMSCs inhibiting NSCLC *in vivo* growth. **(A)** Tumors of nude mice; **(B)** Line graph of the change of tumour volume; **(C)** Statistical graph of tumour weight; **(D)** Expressed LAST2 in tumour tissue analysed via qRT-PCR; **(E)** Expressed E-cadherin, vimentin, and N-cadherin protein detected via western blot; **(F)** Expressed LATS2, p-YAP and YAP protein in tumour tissues detected by western blot; **(G)** Expressed Ki-67 and LATS2 in tumour tissues determined by immunohistochemistry. **p < 0.01 vs exo-NC group, ^##^*p* < 0.01 vs exo-circ_100395 group.

Finally, based on the establishment of xenograft models, it was proved that mass and quantity of the tumour in the exo-circ_100395 group were remarkably dropped as compared to the exo-NC group, which were markedly elevated in the exo-circ_100395 + miR-141-3p and exo-circ_100395 + si-LATS2 groups as compared to the exo-circ_100395 (Figures 7A–C). The results of qRT-PCR and immunohistochemistry showed a significant increase of LATS2 and Ki-67 expressed in exo-circ_100395 group when in comparison with exo-NC group, and a reduction of their expression in the exo-circ_100395 + miR-141-3p and exo-circ_100395 + si-LATS2 groups as compared to the exo-circ_100395 group (Figures 7D–G). In addition, confirmed by western blot that in the exo-circ_100395 group, vimentin and N-cadherin protein expression was relatively decreased and E-cadherin protein expressed in tumour tissues were raised (Figure 7E); meanwhile, YAP protein expression was down-regulated and p-YAP protein expression was up-regulated. However, compared with the exo-circ_100395 group, the changes in the corresponding protein expression above were opposite in the exo-circ_100395 + miR-141-3p and exo-circ_100395 + si-LATS2 groups (Figures 7E,F). Taken together, circ_100395 carried by AMSC-exo is able to contribute to the inhibition of NSCLC *in vivo* growth.

## Discussion

circRNA is classified as one of the non-coding RNA molecules with closed circular structures, which are mainly located in the cytoplasm or stored in exosomes and are expressed in a tissue and disease-specific pattern (Du et al., 2017). Recent research has presented with the fact stating circRNA has close relations with the growth of organisms and diseases (Hsiao et al., 2017). Increasing evidences indicate that circRNA is critical in NSCLC development. For instance, circPTPRA inhibits the EMT and NSCLC cell metastasis by stimulating miR-96-5p (Wei et al., 2019), while circP4HB sponges miR-133a-5p to promote NSCLC invasion and metastasis (Hansen et al., 2013). Additionally, circRNA_101237 promotes NSCLC progression via miRNA-490-3p/MAPK1 axis (Zhang et al., 2020). In this study, we found that circ_100395 was downregulated in lung cancer tissues and cell lines. Moreover, overexpression of circ_100395 could inhibit NSCLC cells proliferation, migration and invasion, and promote cells apoptosis. These results are consistent with the study of Chen et al. (2018).

Serving as sponge role of miRNAs is the main way for circRNA to exert its biological functions (Panda, 2018). MiRNAs is a small non-coding RNA sized around 20 nt is able to target and regulate expressed Mrna (Correia de Sousa et al., 2019). We searched and identified miR-141-3p as the potential target miRNA. And a contrary expression relationship was found between circ_100395 and miR-141-3p in NSCLC tissues. Moreover, luciferase reporter assay and RNA pull down confirmed the relationship. Previous studies revealed that miR-141-3p promotes cancer development. MiR-141-3p, is upregulated in the urine of bladder and prostate cancer individuals and can additionally distinguish benign prostatic hyperplasia accurately from malignant (Ghorbanmehr et al., 2019). In the study of Li et al. (2017) promotion can be done by miR-141-3p for development and stemness of prostate cancer cells by inhibiting kpelrup-like factor-9. MiR-141-3p remarkably raises proliferation, colony formation, invasion and EMT of cancer cells in the cervix to promote tumorigenesis by targeting FOXA2 (Li et al., 2018). Indeed, Li et al. (2019) found that miR-141-3p targets ZFR to inhibit NSCLC progression.

Furthermore, we validated that LATS2 is a target gene of miR-141-3p, and LATS2 expression was regulated by circ_100395/miR-141-3p in NSCLC. LATS2 is a protein that inhibits proliferation and encourages apoptosis in tumour cells. In the previous studies, the down-regulation of LATS2 expression in NSCLC is found linked to aggressive biological behaviour of NSCLC and poor prognosis (Jang et al., 2019). Ye et al. (2017) have proved miR-650 promptly aims for LATS2 for cell proliferation, migration and invasion promotion in NSCLC.

Exosomes are important carriers of circRNAs. Wang et al. have found that in colorectal cancer, exosomes can transport circ_0005963 from chemoresistant cancer cells to chemosensitive cancer cells, and the exosome-delivered circ_0005963 regulates the miR-122-PKM2 pathway to promote glycolysis and induce chemoresistance (Wang et al., 2020). And circRNA secreted from adipocytes can regulate the deubiquitination process in hepatocellular carcinoma, thereby promoting cancer cell growth (Zhang et al., 2019). Collectively, the transmission of signalling molecules by exosomes is an essential link in the process of tumour development. In the results of this study, after treatment with exosomes as delivery vectors, the biological activity of NSCLC cells was inhibited by exo-circ_100395, while they were increased via overexpressed miR-141-3p or knockdown of LATS2. Therefore, it can be inferred that circ_100395 inhibits the biological activity of NSCLC cells by acting as a miR-141-3p sponge.

YAP is a paramount part of Hippo pathway and has a variety of biological functions in the development, homeostasis and regeneration of tissues and organs, and it can also promote EMT to accelerate tumour progression and carcinogenesis (Bourque et al., 2010). Previous study have shown that Yap promotes drug resistance, cancer progression, and metastasis in NSCLC (Janse van Rensburg and Yang, 2016; Rotow and Bivona, 2017; Hsu et al., 2020). In addition, LATS2 can promote YAP phosphorylation, thereby reducing Hippo pathway activity (Shi et al., 2019). Our study showed that exo-circ_100395 reduced Hippo signalling activity, while overexpression of miR-141-3p or knockdown of LATS2 elevated Hippo signalling activity. These results indicate that circ_100395 regulates the development of NSCLC through Hippo signalling by modulating miR-141-3p/LATS2 axis. Similarly, other research pieces have shown circRNA0000140 and circ0001368 can lead to inhibition of oral squamous cell carcinoma and gastric cancer development by inhibiting the Hippo signalling pathway (Liu et al., 2018; Peng et al., 2020).

## Conclusion

Circ_100395 is downregulated in NSCLC tissues and cells. AMSC-exo with overexpression of circ_100395 can increase LATS2 expression by sponging miR-141-3p to inhibit Hippo signalling pathway activity, thus reducing NSCLC progression. This study suggests that overexpression of circ_100395 in AMSC-exo is a new idea for the treatment of NSCLC. However, due to the complex functions of circRNA, further investigation is required to determine whether circ_100395 has other effects on the development of NSCLC.

## Data Availability Statement

The original contributions presented in the study are included in the article/supplementary material, further inquiries can be directed to the corresponding author/s.

## Ethics Statement

The studies involving human participants were reviewed and approved by The First Affiliated Hospital, Zhejiang University School of Medicine (2021-031). Written informed consent for participation was not required for this study in accordance with the national legislation and the institutional requirements. The animal study was reviewed and approved by The First Affiliated Hospital, Zhejiang University School of Medicine (2021-2).

## Author Contributions

CZ and HM: substantial contributions to the conception or design of the work, or the acquisition, analysis, or interpretation of data for the work, drafting the work or revising it critically for important intellectual content. All authors: final approval of the version to be published, agreement to be accountable for all aspects of the work in ensuring that questions related to the accuracy or integrity of any part of the work are appropriately investigated and resolved. All authors contributed to the article and approved the submitted version.

## Conflict of Interest

The authors declare that the research was conducted in the absence of any commercial or financial relationships that could be construed as a potential conflict of interest.
